# Grand Challenges in Pediatric Otolaryngology

**DOI:** 10.3389/fped.2013.00010

**Published:** 2013-05-20

**Authors:** James M. Coticchia, David Cohen, Livjot Sachdeva

**Affiliations:** ^1^Division of Pediatric Otolaryngology, Department of Otolaryngology – Head and Neck Surgery, School of Medicine, Wayne State UniversityDetroit, MI, USA; ^2^Department of Otolaryngology – Head and Neck Surgery, School of Medicine, Wayne State UniversityDetroit, MI, USA

## Introduction

The field of pediatric otolaryngology has undergone significant changes over the past two decades. These changes have resulted in new areas of investigations and challenges. This article will summarize some of the more recent advances and challenges facing this field. Specific focus of this grand challenge will include: (1) Role of biofilms in infectious diseases in otolaryngology, (2) tissue engineering in pediatric airway reconstructive surgery, (3) minimally invasive surgical techniques, and (4) advances in molecular biology of sensorineural hearing loss (SNHL).

## Biofilms as Unique Model for Chronic and Recurrent Infections in Otolaryngology

The burden of infectious diseases in several clinical problems encountered in pediatric otolaryngology is quite extensive. Though previously not well defined, the evidence to support biofilms as a pathophysiologic model in otolaryngology has increased remarkably over the last decade. Biofilms have been implicated in the pathogenesis of otitis media (OM), chronic rhinosinusitis (CRS), chronic tonsillitis, cholesteatoma, recurrent tracheitis, and cochlear implant infections.

The initial implication of biofilm phenotypes in acute OM (AOM) and OM with effusion (OME) was postulated following the observation fact that many patients with middle ear effusion were culture negative. Furthermore, several studies demonstrated that a significant portion of these effusions did in fact have bacterial mRNA or were PCR positive for bacterial DNA (1). These observations were paramount and suggested that biofilms may play a role in OM. Further research demonstrated that the three predominant causative agents for OM, *Streptococcus pneumoniae*, *Haemophilus influenzae*, and *Moraxella catarrhalis*, form biofilms in both *in vitro* and *in vivo* settings (2). This evidence, along with several investigations, would suggest that the primary pathogens for OM are capable of forming biofilms (2, 3). This is an important observation as bacteria in biofilm phenotypes can survive at antibiotic concentrations >2000 times the minimum inhibitory concentration (MIC) (4, 5).

The clinical importance of biofilm formation by known middle ear pathogens (MEPs) was also demonstrated by an animal model that closely parallels the human disease state. The model utilized superinfection of Influenza A and *S. pneumoniae* inoculation via the nasopharynx. Approximately 37% of these animals developed AOM and scanning electron microscopy (SEM) imaging identified robust biofilms in the nasopharynx, eustachian tube, and ME mucosa of these infected animals (6).

In regard to CRS, the chronic and medically refractive nature of the disease lends itself naturally to a biofilm model. Both human and rabbit models of sinusitis have repeatedly demonstrated the capability of infectious agents to form biofilms. In a critical study, Ramadan et al. analyzed tissue samples from patients with CRS under SEM and successfully showed that every sample had biofilms present in various phases of proliferation (7). Further studies confirmed that the three most common causative bacterial agents readily formed biofilms in respiratory epithelium of patients with CRS.

### Future challenges

- Development of non-invasive imaging techniques like optical coherence tomography and high frequency ultrasound to identify biofilms inside the human body (8, 9).
- Characterizing the role of host surface receptors in allowing initial attachment of biofilm forming pathogens.
- Defining the role of viral co-infection in altering respiratory epithelial surface to allow attachment and development of biofilms.
- Studying the role of host immune response to the formation of biofilms.
- Elucidating the environmental factors that favor development and/or degradation of biofilms. Recent studies have alluded to certain EPS lyases that are produced in response to specific environmental triggers such as oxygen depletion and can enzymatically degrade the EPS matrix releasing the cells from the biofilm (10).

## Airway Reconstruction Utilizing Tissue Engineering Techniques

Tissue engineering is an interdisciplinary field where engineering and life science intersect with the hope of creating a biological substitute for human organs and tissues. Although airway reconstructive surgery arose in the late twentieth century, the rapid development and complexity of bioengineering and tissue regeneration has significantly shaped the current approach to airway reconstruction today.

Surgical management of long-segment tracheal stenosis in children by its very nature is very complex and challenging and has been evolving over the last two decades with patch (graft) tracheoplasty and slide tracheoplasty as the most common surgical techniques. Although the tissue grafts employed in patch tracheoplasty re-epithelialize rapidly, the eventual outcome of the surgery can be complicated by restenosis arising from granulation or shrinkage of the graft thus requiring re-intervention. However, the experience with this technique has been reported in literature with varying degrees of success (11, 12). Fanous et al. reported successful long term outcomes for pericardial tracheoplasty performed on 26 patients with long-segment tracheal stenosis with five hospital deaths, none from airway obstruction, and two re-interventions (13). More recently, slide tracheoplasty with cardiopulmonary bypass support has shown encouraging short-term outcomes with early extubation post-op and shorter hospital stays for patients. Several authors have reported successful outcomes for long-segment stenosis using this technique with lower mortality rates ranging from 10 to 30%, fewer post-op complications and re-interventions as compared to other techniques (14–21). As with patch tracheoplasty, varying degrees of success have been reported with slide tracheoplasty with Manning et al. demonstrating lower rates of complications contrasting with the less encouraging experience of Wright et al. (22, 23).

Owing to the complexity of airway surgical management in children and varying degrees of successes with the current surgical techniques, the quest for the development of a novel allograft or biological implant for airway reconstruction is only logical. There are several limitations to creating novel implants in the airway reconstructive setting. These include life-threatening complications, small sample sizes, and a lack of human subjects due to the precarious nature of airway surgery. Such complications, including migration, dislodgement, infection, obstruction, adjacent site stenosis or granulation, and necrosis, have the potential for significant host morbidity and/or mortality. In this way, the development of such an implant has been exceedingly difficult. For this reason, no clinically convincing tracheal replacement method or implant currently exists.

Belsey described the theoretical tracheal implant in the 1950s as a laterally rigid but longitudinally flexible, biocompatible, non-immunogenic, bacterial resistant, airtight stent that promotes respiratory epithelialization (24). Early scientists were thus disillusioned when they believed the implant to be as simple as developing a tubular cartilaginous structure. Later, animal models confirmed the importance of respiratory epithelialization in preventing granulomatous tissue formation and promoting cilia formation for functionality (25). Initial research efforts were largely tissue scaffolds aimed at guiding innate tissues for quicker regeneration. Shimizu and his colleagues were the first to use a synthetic scaffold consisting of Marlex mesh tube covered in a collagen sponge made from porcine dermal collagen as a tracheal implant, which was unsuccessful (26). The major limitation of acellular scaffolding implants is the potential for incomplete re-epithelialization, which may lead to biofilm formation, granulation tissue growth, or airway stenosis from cicatricial scar formation (27). Similarly, an animal study by Weidenbacher et al. demonstrated that when using a neo-tracheal tubular implant, all animals eventually developed cicatricial scar formation and had to be sacrificed (28). Therefore, the importance of epithelialization was confirmed, and research shifted toward tissue-engineered implants.

Tissue engineering involves the implantation of stem-cells onto bioscaffolding matrices. In these tissue models, functionality and epithelialization are aided through the use of stem-cells, which are believed to hasten the successful incorporation of such implants. While some studies have demonstrated the capability to seed mesenchymal stem-cells onto bioscaffolds to produce viable cardiac myocyte regeneration, the harvest and proliferation of human respiratory epithelial cells has proved more challenging (29). Currently, there is still no widely accepted seeding method for these cells (25). In addition to lacking a reliable source for respiratory stem-cells, further challenge rests in the structure of the human trachea itself. Between the epithelial and cartilaginous layers is a submucosal layer that is filled with a dense capillary network (30). Some scientists, such as Tan et al., hypothesize that it is the incapability of the implant to simulate this permeable quality that leads to such poor rates of successful allograft implantation. Others argue that epithelialization of the implant is key to successful implantation. Likely, it is a combination of these factors, as well as additional factors that have not been uncovered, which would ultimately lead to improved tissue-engineered tracheal implantation. In this way, the tissue-engineered trachea is still far away from clinical application. However, it remains a topic of interest due to its vast potential if such an implant is able to be designed.

### Future challenges

- Tissue-engineered construct for laryngotracheal reconstruction
- Tissue-engineered construct for long-segment tracheal stenosis
- Utility of augmented cadaveric fragment in tracheal reconstruction

## Minimally Invasive Techniques and Robotic Surgery

Numerous fields have begun to embrace minimally invasive surgical approaches. These include laparoscopic surgery, endoscopic surgery approach for neurosurgical procedures, endoscopic management of mediastinal lesions to name a few. The advantages of minimally invasive procedures include decreased cosmetic scars, less dissection, and decreased morbidity. In addition to endoscopic procedures, robotic procedures offer many of the same advantages (31). Some recent areas of investigation include robotic assisted airway surgery, endoscopic airway surgery, endoscopic and robotic assisted thyroid surgery, and the potential for sentinel lymph nodes biopsy (32). Current applications in pediatric otolaryngology include endoscopic approach for juvenile nasal angiofibromas and endoscopic approaches for skull base tumors in children (33). In addition, other areas of investigation include robotic assisted tympanomastoid surgery and image guidance endoscopic management of congenital problems including choanal atresia and canal atresia.

### Future challenges

- Develop smaller instruments and novel approaches for endoscopic robotic assisted airway surgery
- Develop novel techniques for endoscopic neck biopsies and management of congenital neck mass
- Develop a wider array of instruments and novel approaches for image guided endoscopic choanal atresia surgery
- Utilize robotic approach and image guidance for pediatric mastoid surgery and cochlear implant surgery

## Molecular Biology of Auditory Regeneration in Snhl

Loss of inner ear (IE) functionality and balance disorders due to hair cell damage can occur through ototoxic, acoustic, environmental, or chemical damage as well as aging or genetic conditions. Restoration of IE functionality through hair cell regeneration was not even considered a possibility until the discovery of hair cell regeneration in avian IE. While non-mammals mount an active regenerative response to hair cell damage via proliferation and differentiation of supporting cells, auditory regeneration in mammalian IE is not as pronounced since most supporting cells maintain mitotic quiescence. However, investigations to study the role of protein regulation, growth factors, and molecular signaling pathways in the regenerative response in non-mammals have opened a plethora of opportunities to manipulate and study the response in mammals.

Several key regulators have been identified in non-mammals that can stimulate or inhibit proliferation of supporting cells. Insulin-like growth factor 1 (IGF-1) has been shown to be upregulated in avian vestibular epithelium post hair cell damage while fibroblast growth factor 2 (FGF-2) has been shown to inhibit cell proliferation (34, 35). Expression of actin-interacting protein WD40 and fibroblast growth factor receptor 3 (FGFR3) has been shown to be upregulated in the supporting cells in non-mammals that exhibit hair cell regeneration (36, 37). Studies by Bermingham et al. (1999) have demonstrated the role of basic helix-loop-helix transcription factor like mammalian atonal homolog 1 (Math1) in promoting the differentiation of supporting cells into hair cells (38). However, *in vivo* studies by several investigators to manipulate the expression of Math1 in the organ of Corti in mammals have revealed mixed evidence with respect to regenerative response and functional recovery (39–43).

### Future challenges

- Gene expression profiling to study regulation of cell proliferation in auditory and vestibular sensory epithelia
- Targeted gene delivery and expression and potential gene therapy using adenovirus technology (44)
- Using stem cell technology to induce cells to proliferate and differentiate into hair cells (45)

## Conclusion

Pediatric otolaryngology was born out of a need for specialists to treat and manage congenital complications in infants and children. Since its inception, this field has made rapid advances with innovative technological breakthroughs, scientific discoveries, and therapeutic interventions – some of which have been briefly discussed in this article. However, as outlined above, there are some great challenges ahead of us and it’s imperative that we ride on our recent success and look to extend the current boundaries. With science and medicine becoming more and more interdisciplinary, we are poised to make rapid advances and are placed in an excellent position to overcome current challenges and discover new ones.
